# Implications of accounting for marker-based population structure in the quantitative genetic evaluation of genetic parameters related to growth and wood properties in Norway spruce

**DOI:** 10.1186/s12863-024-01241-x

**Published:** 2024-06-14

**Authors:** Haleh Hayatgheibi, Henrik R. Hallingbäck, Sven-Olof Lundqvist, Thomas Grahn, Gerhard Scheepers, Peter Nordström, Zhi-Qiang Chen, Katri Kärkkäinen, Harry X. Wu, M. Rosario García-Gil

**Affiliations:** 1 Department of Forest Genetics and Plant Physiology, Swedish University of Agricultural Sciences (SLU), Umeå, Sweden; 2 Forestry Research Institute Sweden (Skogforsk), 75183 Uppsala, Sweden; 3 IIC, Stockholm, Sweden; 4 RISE AB, Stockholm, Sweden; 5 RISE AB, Växjö, Sweden; 6 RISE AB, Umeå, Sweden; 7 Natural Resources Institute Finland (LUKE), OULU, Finland; 8 Beijing Advanced Innovation Centre for Tree Breeding By Molecular Design, Beijing Forestry University, Beijing, China; 9 Black Mountain Laboratory, CSIRO National Collection Research Australia, Canberra, Australia

**Keywords:** Norway spruce, Population structure, Wood properties, Cross-validation, Prediction accuracy

## Abstract

**Background:**

Forest geneticists typically use provenances to account for population differences in their improvement schemes; however, the historical records of the imported materials might not be very precise or well-aligned with the genetic clusters derived from advanced molecular techniques. The main objective of this study was to assess the impact of marker-based population structure on genetic parameter estimates related to growth and wood properties and their trade-offs in Norway spruce, by either incorporating it as a fixed effect (model-B) or excluding it entirely from the analysis (model-A).

**Results:**

Our results indicate that models incorporating population structure significantly reduce estimates of additive genetic variance, resulting in substantial reduction of narrow-sense heritability. However, these models considerably improve prediction accuracies. This was particularly significant for growth and solid-wood properties, which showed to have the highest population genetic differentiation (Q_ST_) among the studied traits. Additionally, although the pattern of correlations remained similar across the models, their magnitude was slightly lower for models that included population structure as a fixed effect. This suggests that selection, consistently performed within populations, might be less affected by unfavourable genetic correlations compared to mass selection conducted without pedigree restrictions.

**Conclusion:**

We conclude that the results of models properly accounting for population structure are more accurate and less biased compared to those neglecting this effect. This might have practical implications for breeders and forest managers where, decisions based on imprecise selections can pose a high risk to economic efficiency.

**Supplementary Information:**

The online version contains supplementary material available at 10.1186/s12863-024-01241-x.

## Background

It is well known that the geographic ranges of tree species have expanded and contracted several times during glacial and interglacial periods [1]. The contraction phase led to the isolation between refugial area and substantial differentiation between populations [2]. During the postglacial period, as climatic conditions successively improved, expansions formed the secondary contacts between the migrating fronts, resulting in introgression, and in Fennoscandia, establishment of steep latitudinal and longitudinal clines for adaptive traits [3, 4]. Indeed, the new biotic and abiotic factors encountered during the glaciation cycles, resulted in new adaptations [5]. Individual phenotypes are thus the result of a dynamic interplay between evolutionary and demographic processes [6, 7], which has resulted in the formation of specific tree species characteristics that may uniquely enable them to survive environmental changes.

Rapid changes in climatic conditions trigger new selective pressures on existing populations, which need to rapidly adapt to these changing conditions [8, 9]. Migration to more suitable habitats and phenotypic plasticity are regarded as short-term responses to these changes [10]. Nevertheless, long-term responses require adaptive capacity, which depends on the level of genetic variation, upon which selection can act [11]. Unravelling the factors underlying the adaptive traits is of particular interest for evolutionary biologists, forest tree breeders as well as conservation geneticists [12], and to successfully achieve this; one useful approach is to investigate how phenotypic variation is partitioned within and among populations [13].

Common garden testing of populations, so-called provenance tests, include broad genetic material representing a large proportion of a species’ natural distribution. This provides a great opportunity to dissect genetic variation within a species and to determine the responses of populations to changing climate conditions through phenotypic plasticity and genetic selection to the changes [14]. The presence of genetic variation within a population implies that the trait variation is heritable, while among population variation (e.g., Q_ST_) can be the result of population demography. In general, these estimates provide important information on the ability of populations to respond to selection [15]. For instance, narrow-sense heritability is a key population parameter which is defined as the proportion of phenotypic variation that is due to additive genetic variance, upon which selection can act [11], and therefore is widely used in genetic improvement schemes [12].

Norway spruce (*Picea abies* (L.) H. Karst.) is a dominant conifer species of major economic importance in northern Europe. In Nordic countries, Norway spruce is an essential source of raw material for the forest-based industry, mainly used either for construction purposes or for pulp production [16]. As such, the transfer and trade of Norway spruce seeds and seedlings from other regions and countries was extensive in Southern Sweden already during the 19^th^ century because foresters observed that such materials were more productive than local provenances. In the 1940s, a large-scale Norway spruce breeding program was initiated in Sweden, by selecting trees with superior phenotype (plus-trees) for first-generation seed orchards [17]. And later, systematic genetic testing in the field confirmed that stands planted with materials of foreign origin indeed were more productive than the local provenances and appeared well adapted to the conditions in southern Sweden [18]. Therefore, to increase the stand productivity and overcome seed deficits, large quantities of Norway spruce seeds from central and south-eastern Europe were imported to southern parts of Sweden both before and during the age of tree breeding [19]. The geographic variation in the genetic make-up of introduced trees implies a strong population and admixture structure of base materials in the breeding program. Recently, genome-wide data analysis, utilizing the base populations of the Swedish breeding program, has revealed a more complex demographic history of the species, where three main genetic clusters had been described in Norway spruce: a northern domain in Fennoscandia and two southern domains in the Alps and Carpathians [20, 21]. Other studies of recurrent demographic migrations, followed by secondary interactions indicated a fragmentation of Norway spruce into seven genetic clusters [22–24]: Northern Fennoscandia (NFE), Central and South Sweden (CSE), Russia-Baltics (RusBal), Northern Poland (NPL), Central Europe (CEU), Alpine (ALP), and Carpathian (ROM) domains. In southern Sweden, a hybrid between CSE and ALP (CSE-ALP) has also recently been discovered [7].

A successful genetic improvement program depends on reliable estimates of additive genetic variance, narrow-sense heritability, and additive genetic correlations between traits [25]. Since domestication of most of forest trees is still in its early stage, the understanding of the effect of geographical differences among the plus-trees greatly affects the results of genetic evaluations, and in turn, subsequent generations of breeding. Although forest geneticists have widely adopted provenances to account for population differences in their improvement schemes, the historical records of such imported materials might not be very precise or well-aligned with the genetic clusters derived from advanced molecular techniques.

Genetic parameters for growth and wood properties of Norway spruce have been extensively studied [16, 26, 27]. However, the effect of genetic clusters (hereafter population structure) in genetic evaluations was not explicitly estimated. Therefore, the main objective of this study is to elucidate the effect of population structure, retrieved from genome-wide DNA markers, on genetic parameter estimates, fitted as a fixed effect or excluded using two mixed-linear model alternatives. More specifically, this paper addresses the following objectives: 1) to evaluate differences in phenotypic and genetic performances of Norway spruce populations for growth and wood properties 2) to quantify the level of population differentiation (Q_ST_), narrow-sense heritability (${h}^{2}$), and additive genetic correlations (trade-offs) between growth and wood properties 3) To compare the predictive ability (PA) and prediction accuracy (ACC) of models using random cross validation. The analysis was done using a large dataset of 5,666 20-year-old trees from 524 half-sib families. Such investigation has practical implications for breeders and forest managers where, decisions based on imprecise selection of parameter estimates or wrong assumptions can pose a high risk to economic efficiency.

## Result

### Comparison of phenotypic and genetic performances across populations

Two different mixed models (model-A and model-B) were tested to investigate the effect of population structure fitted as a fixed effect on genetic parameter estimates for different growth and wood properties. In models accounting for population structure (model-B), the effect of population structure was highly significant for all studied traits (*p* < 0.001), except for CELL and HEM (Table S1).

**Fig.1:**
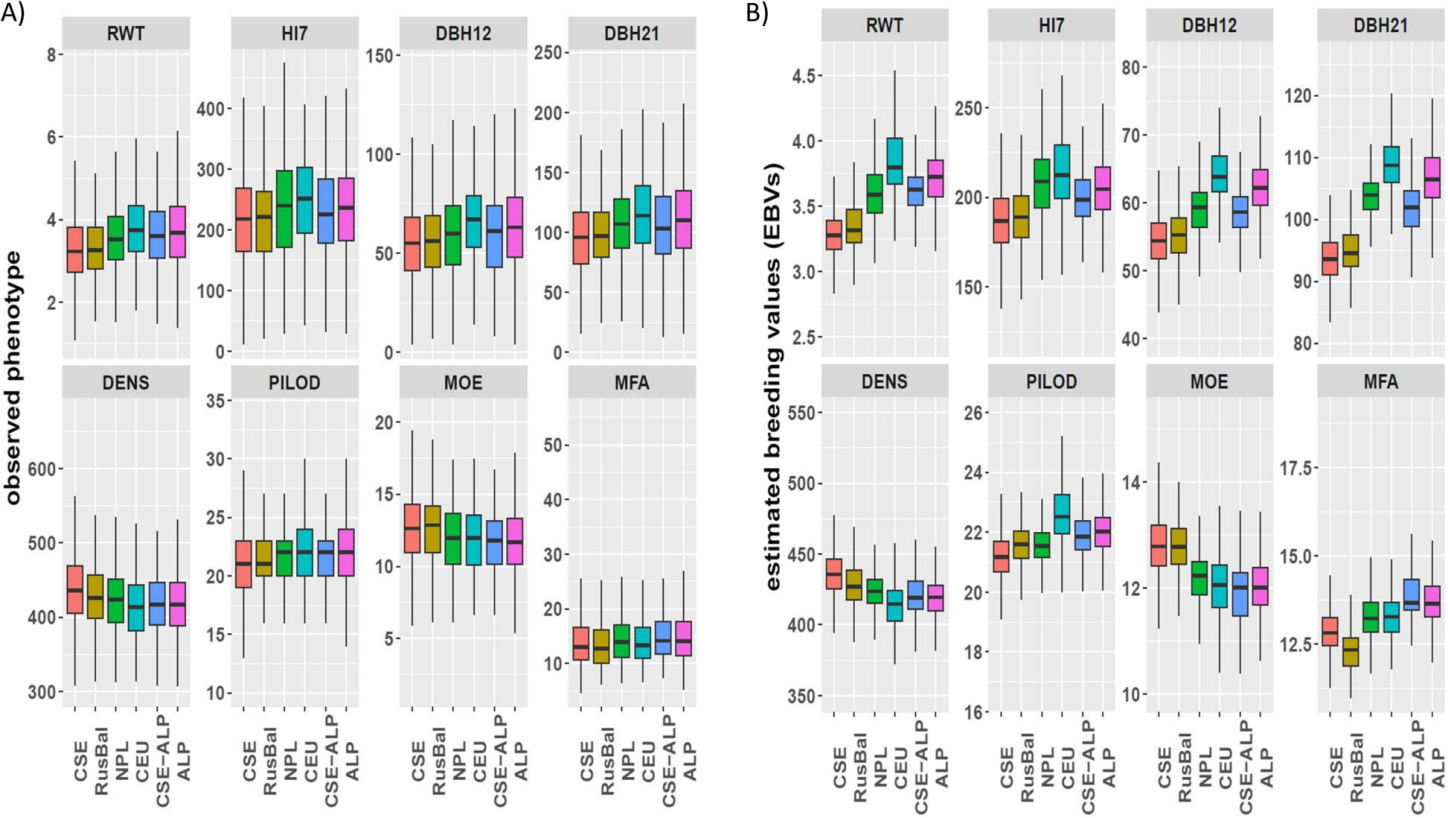
Boxplots of phenotypic (**A**) and genetic (**B**) (EBVs of individuals obtained based on model-B) performances of Norway spruce populations measured for growth and solidwood properties. Populations ordered by decreasing latitude from left to right on the x-axis. Central and South Sweden (CSE, red), Russia-Baltic (RusBal, olivegreen), Northern Poland (NPL, green), Central Europe (CEU, light blue), hybrids between CSE and ALP (CSE-ALP, blue), and Alps (ALP, pink). Trait abbreviations are explained in Table 6

The performances of phenotypes and EBVs (using model-B only) across populations assessed for growth and solidwood properties are shown in Fig. 1. Such assessments for other studied traits are further included in supplementary materials (Fig. S1). Overall, populations showed greater differentiation based on the EBVs compared to the phenotypic values, suggesting that the variations among populations are primarily genetic rather than environmental. Nevertheless, in both performance categories, southern populations, such as ALP, CEU and NPL, were the most productive ones (higher RWT, HI7, DBH12, DBH21, TRadW, TTangW, MFA, and NUMRES). In contrast, their solid wood properties (DENS, MOE, TWTH) were lower than those of northern populations, such as CSE. In general, trees of CEU origin had the highest growth-related properties, while they had the lowest density-related properties. Such trends were particularly noticeable for EBVs. Trees of RusBal origin performed similarly to those of CSE origin, especially in terms of growth, whereas they had slightly lower DENS, FWT, FC and higher MOE (Fig. 1 and Fig. S1). No significant differences were observed among populations for chemical properties, except for LIG which was slightly higher for southern populations than CSE and RusBal (Fig. S1).

**Fig. 2:**
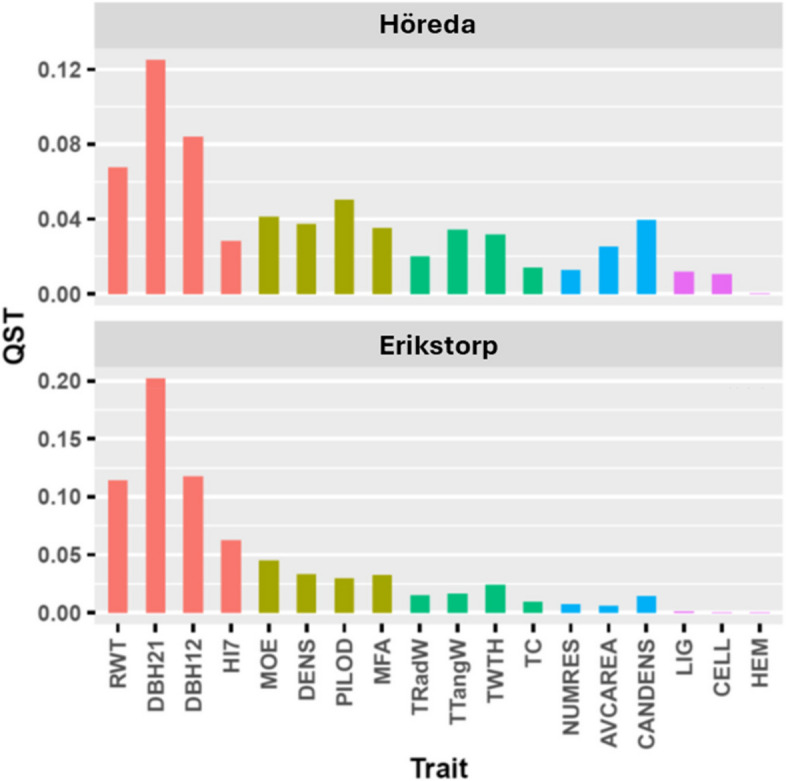
Q_ST_-estimates for annual ring-width (RWT), diameter measured at ages 21 and 12 (DBH21 and DBH12, respectively), height measured at age 7 (HI7), modulus of elasticity (MOE), density (DENS), pilodyn (PILOD), microfibril angle (MFA), tracheid radial width (TRadW), tangential tracheid width (TTangW), tracheid wall thickness (TWTH), tracheid coarseness (TC), total number of resin canals (NUMRES), average area of resin canals (AVCAREA), resin Canal density (CANDENS), lignin (LIG), Cellulose (CELL) and hemicellulose (HEM) content at two progeny trials Höreda and Erikstorp

Generally, Q_ST_ values were higher for growth traits with the greatest estimates obtained for DBH21 (~ 0.13 in Höreda and 0.20 in Erikstorp), which represent the highest genetic differentiation among populations, while Q_ST_ values for chemical wood properties were nearly zero (Fig. 2). Q_ST_ values for solidwood properties (0.03 to 0.05 in both trials) were generally higher than those observed for tracheid and resin properties with the greatest value obtained for TTWT and TTangW at ~ 0.03 (Fig. 2).

### Quantitative genetic parameter estimates

#### Narrow-sense heritability estimates

The most considerable difference between model-A and model-B, in terms of genetic parameter estimates, was the notable reduction of the additive genetic variance and the consequent reduction of narrow-sense heritabilities (${h}^{2}$) observed in model-B, particularly for growth (Tables 1 and 2). For example, in Höreda, the additive genetic variance estimates for DBH21 and RWT were 47% and 63% of the estimates derived from model-A, respectively. In Erikstorp, such proportions were 36% and 48%, respectively. Overall, ${h}^{2}$ was reduced by about 50% in Höreda and 65% in Erikstorp for DBH21. Similarly, ${h}^{2}$ for solidwood and some of tracheid properties were lower based on model-B. For instance, estimates for DENS, MOE, MFA, and TWTH decreased by 19.8%, 23.4%, 15.5%, and 17.5%, respectively in Höreda, while in Erikstorp, they decreased by 18.2%, 21.4%, 11.4%, and 15%, respectively. Nevertheless, reductions in ${h}^{2}$ based on model-B were negligible for resin properties and ${h}^{2}$ estimates for chemical properties were very similar in both models.

**Table 1:** Estimated genetic parameters for growth and wood properties in trial Höreda based on two mixed-model approaches (model-A and model-B) excluding and including a fixed effect of population structure, respectively

Höreda							
model-A				model-B			
Trait	${\sigma }_{A}^{2}$	${\sigma }_{e}^{2}$	${h}^{2}$	Trait	${\sigma }_{A}^{2}$	${\sigma }_{e}^{2}$	${h}^{2}$
DBH12	92.578 (12.996)	203.693 (12.117)	0.312 (0.042)	DBH12	55.205 (10.737)	232.319 (10.874)	0.192 (0.036)
DBH21	308.773 (42.882)	599.653 (39.694)	0.339 (0.045)	DBH21	145.287 (33.176)	723.673 (34.254)	0.167 (0.037)
HI7	1537.417 (194.117)	2488.117 (175.284)	0.381 (0.045)	HI7	1283.008 (180.207)	2679.159 (167.396)	0.323 (0.043)
RWT	0.456 (0.049)	0.107 (0.041)	0.809 (0.076)	RWT	0.288 (0.039)	0.234 (0.034)	0.551 (0.069)
PILOD	3.759 (0.462)	2.174 (0.401)	0.633 (0.070)	PILOD	2.776 (0.406)	2.913 (0.366)	0.487 (0.066)
DENS	1414.590 (144.142)	151.082 (119.165)	0.903 (0.077)	DENS	1069.350 (123.840)	410.860 (105.611)	0.722 (0.074)
MOE	2.196 (0.325)	2.382 (0.295)	0.479 (0.066)	MOE	1.631 (0.292)	2.808 (0.275)	0.367 (0.063)
MFA	3.703 (1.034)	14.203 (1.045)	0.206 (0.057)	MFA	3.105 (1.003)	14.651 (1.028)	0.174 (0.055)
TRadW	2.669 (0.296)	0.801 (0.250)	0.769 (0.074)	TRadW	2.330 (0.277)	1.055 (0.238)	0.688 (0.073)
TtangW	1.015 (0.159)	1.290 (0.146)	0.440 (0.065)	TtangW	0.904 (0.153)	0.897 (0.148)	0.397 (0.064)
TWTH	0.024 (0.002)	0.011 (0.002)	0.681 (0.072)	TWTH	0.019 (0.002)	0.015 (0.002)	0.562 (0.069)
TC	406.816 (68.393)	613.229 (63.996)	0.398 (0.064)	TC	383.576 (67.456)	631.353 (63.482)	0.377 (0.063)
NUMRES	33.566 (7.761)	87.851 (7.613)	0.276 (0.062)	NUMRES	30.420 (7.628)	90.322 (7.548)	0.251 (0.062)
AVCAREA	12,238.63 (2250.014)	20,698.93(2127.098)	0.371 (0.065)	AVCAREA	11,354.158 (2208.23)	21,369.810 (2442.938)	0.346 (0.065)
CANDENS	52.721 (11.963)	149.329 (11.733)	0.260 (0.057)	CANDENS	39.792 (10.931)	159.102 (11.055)	0.200 (0.054)
CELL	0.236 (0.081)	0.715 (0.081)	0.249 (0.084)	CELL	0.228 (0.081)	0.721 (0.081)	0.240 (0.084)
HEM	0.031 (0.013)	0.134 (0.014)	0.189 (0.082)	HEM	0.030 (0.013)	0.135 (0.014)	0.183 (0.082)
LIG	0.103 (0.026)	0.186 (0.025)	0.357 (0.087)	LIG	0.097 (0.025)	0.191 (0.024)	0.337 (0.087)

${\sigma }_{A}^{2}$ additive genetic variance, ${\sigma }_{e}^{2}$ residual variance, ${h}_{i}^{2}$ narrow-sense heritability estimates (standard error of estimates in parenthesis)

**Table 2:** Estimated genetic parameters for growth and wood properties in trial Erikstorp based on two mixed-model approaches (model-A and model-B) excluding and including a fixed effect of population structure, respectively

Erikstorp							
model-A				model-B			
Trait	${\upsigma }_{\text{A}}^{2}$	${\upsigma }_{\text{e}}^{2}$	${\text{h}}^{2}$	Trait	${\upsigma }_{\text{A}}^{2}$	${\upsigma }_{\text{e}}^{2}$	${\text{h}}^{2}$
DBH12	144.117 (18.831)	235.604 (17.106)	0.379 (0.046)	DBH12	72.496 (14.085)	289.447 (14.229)	0.200 (0.038)
DBH21	197.469 (37.468)	555.135 (36.588)	0.262 (0.048)	DBH21	71.435 (26.931)	648.502 (30.298)	0.099 (0.037)
HI7	1326.204 (185.363)	2730.078 (172.451)	0.326 (0.043)	HI7	883.436 (153.294)	3057.163 (152.214)	0.224 (0.038)
RWT	0.319 (0.048)	0.299 (0.043)	0.515 (0.073)	RWT	0.154 (0.038)	0.422 (0.037)	0.267 (0.065)
PILOD	2.931 (0.539)	4.476 (0.507)	0.395 (0.069)	PILOD	2.592 (0.524)	4.739 (0.499)	0.353 (0.068)
DENS	877.921 (113.879)	449.547 (99.470)	0.661 (0.077)	DENS	694.502 (103.063)	587.734 (92.554)	0.541 (0.074)
MOE	1.931 (0.350)	2.819 (0.328)	0.406 (0.070)	MOE	1.481 (0.323)	3.155 (0.311)	0.319 (0.067)
MFA	4.426 (1.393)	17.583 (1.412)	0.201 (0.062)	MFA	3.898 (1.363)	17.965 (1.394)	0.178 (0.061)
TRadW	1.712 (0.227)	0.935 (0.199)	0.646 (0.078)	TRadW	1.590 (0.220)	1.025 (0.194)	0.608 (0.077)
TtangW	0.951 (0.151)	1.005 (0.137)	0.486 (0.073)	Ttang	0.897 (0.148)	1.045 (0.136)	0.461 (0.072)
TWTH	0.016 (0.002)	0.013 (0.002)	0.540 (0.073)	TWTH	0.013 (0.002)	0.015 (0.002)	0.459 (0.071)
TC	316.083 (56.237)	454.171 (52.736)	0.410 (0.069)	TC	299.292 (55.570)	466.891 (52.340)	0.390 (0.069)
NUMRES	42.782 (11.883)	101.805 (11.647)	0.295 (0.080)	NUMRES	41.332 (11.769)	102.707 (11.565)	0.286 (0.079)
AVCAREA	7732.33 (2437.293)	22,818.36 (2423.491)	0.253 (0.078)	AVCAREA	7999.531 (2471.36)	22,602.401 (2442.938)	0.261 (0.079)
CANDENS	48.423 (11.586)	103.229 (11.154)	0.319 (0.074)	CANDENS	44.767 (11.611)	106.004 (11.265)	0.296 (0.074)
CELL	0.267 (0.090)	0.721 (0.089)	0.270 (0.089)	CELL	0.279 (0.091)	0.712 (0.089)	0.281 (0.090)
HEM	0.036 (0.011)	0.093 (0.011)	0.282 (0.090)	HEM	0.038 (0.012)	0.092 (0.011)	0.293 (0.092)
LIG	0.074 (0.030)	0.267 (0.030)	0.216 (0.087)	LIG	0.077 (0.030)	0.264 (0.030)	0.225 (0.087)

${\sigma }_{A}^{2}$ additive genetic variance, ${\sigma }_{e}^{2}$ residual variance, ${h}_{i}^{2}$ narrow-sense heritability estimates (standard error of estimates in parentheses)

#### Evaluation of models using k-fold Cross-validation

Predictive ability (PA) and prediction accuracy (ACC) of model-A and model-B were estimated through 5-fold cross validation. Despite the significantly lower additive genetic variances and ${h}^{2}$ estimates obtained in model-B, this model resulted in higher PA and ACC for all studied traits in both trials, except for the marginally lower estimates obtained for chemical properties in Erikstorp (Table 3). The improvements in PA and ACC were particularly noticeable for growth, where ACC increased by 44%, 35%, and 26% for DBH21, DBH12, and RWT, respectively in Höreda and by 68%, 42%, and 44%, respectively in Erikstorp. Correspondingly, improvements in ACC for DENS, MOE, and TWTH were 17%, 25%, and 17%, respectively in Höreda and by 17%, 31%, and 15%, respectively in Erikstorp (Table 3).

**Table 3:** Trait predictive ability (PA) and prediction accuracy (ACC) based on model-A and model-B in two trials Höreda and Erikstorp. (standard errors of the estimates in the parentheses)

Höreda					Erikstorp					
Traits	Predictive Ability (PA)		Prediction Accuracy (ACC)		Traits	Predictive Ability (PA)			Prediction Accuracy (ACC)	
	model-A	model-B	model-A	model-B		model-A	model-B		model-A	model-B
DBH21	0.171 (0.007)	0.211 (0.009)	0.292	0.523	DBH21	0.114 (0.014)	0.203 (0.015)		0.220	0.682
DBH12	0.146 (0.008)	0.173 (0.008)	0.271	0.418	DBH12	0.194 (0.009)	0.244 (0.011)		0.306	0.529
HI7	0.156 (0.008)	0.165 (0.008)	0.259	0.298	HI7	0.173 (0.009)	0.201 (0.013)		0.291	0.399
RWT	0.282 (0.014)	0.312 (0.011)	0.307	0.413	RWT	0.209 (0.015)	0.277 (0.010)		0.282	0.504
DENS	0.310 (0.011)	0.329 (0.009)	0.325	0.391	DENS	0.247 (0.007)	0.268 (0.008)		0.294	0.354
PILOD	0.216 (0.014)	0.240 (0.017)	0.279	0.360	PILOD	0.143 (0.01)	0.159 (0.009)		0.221	0.265
MOE	0.192 (0.019)	0.221 (0.018)	0.293	0.393	MOE	0.151 (0.009)	0.194 (0.013)		0.229	0.332
MFA	0.097 (0.010)	0.114 (0.017)	0.220	0.285	MFA	0.075 (0.009)	0.111 (0.015)		0.162	0.257
TTangW	0.184 (0.013)	0.199 (0.014)	0.276	0.318	TTangW	0.211 (0.018)	0.217 (0.018)		0.301	0.318
TRadW	0.286 (0.008)	0.299 (0.010)	0.326	0.363	TRadW	0.248 (0.027)	0.251 (0.023)		0.318	0.332
TWTH	0.247 (0.008)	0.266 (0.006)	0.294	0.352	TWTH	0.209 (0.010)	0.225 (0.009)		0.267	0.313
TC	0.168 (0.009)	0.176 (0.004)	0.254	0.274	TC	0.173 (0.023)	0.170 (0.020)		0.253	0.257
NUMRES	0.115 (0.019)	0.114 (0.014)	0.213	0.221	NUMRES	0.110 (0.09)	0.122 (0.020)		0.190	0.211
AVCAREA	0.141 (0.013)	0.150 (0.017)	0.224	0.246	AVCAREA	0.083 (0.015)	0.082 (0.015)		0.167	0.167
CANDENS	0.088 (0.021)	0.122 (0.027)	0.178	0.282	CANDENS	0.117 (0.010)		0.125 (0.006)	0.210	0.233
LIG	0.118 (0.008)	0.132 (0.009)	0.190	0.218	LIG	0.061 (0.017)		0.055 (0.019)	0.126	0.112
CELL	0.081 (0.014)	0.095 (0.017)	0.156	0.184	CELL	0.063 (0.016)		0.050 (0.016	0.118	0.092
HEM	0.077 (0.015)	0.078 (0.010)	0.172	0.175	HEM	0.083 (0.015)		0.051 (0.007	0.159	0.179

#### Additive genetic correlations (trade-offs) and genetic correlations between sites (G × E)

A subset of additive genetic correlations (${r}_{a}$) among different traits, based on both models, are presented in Table 4, whereas the complete set is given in supplementary materials (Table S2). Additionally, the magnitude of ${r}_{a}$ was assessed separately for the two main populations underlying this study (CSE and ALP) and a subset of their results are shown in Table 5. As expected, MOE and DENS were negatively associated with growth traits across the models, ranging from -0.66 (between MOE and DBH21) to -0.74 (between RWT and DENS) based on model-A. However, the magnitude of correlation estimates was lower based on model-B, ranging from -0.52 (between MOE and DBH21) and -0.62 (between RWT and DENS), corresponding to about 21% and 16% reduction of growth and wood properties trade-offs, compared to model-A (Table 4). Additionally, NUMRES and TRadW, which are positively associated with growth, showed negative genetic correlation with DENS, ranging from -0.50 to -0.65, respectively based on model-A, and from -0.42 to -0.58, respectively based on model-B. Additionally, ${r}_{a}$ between LIG and DENS was negative in both models, with a slight reduction (~ 3%) of the correlation based on model-B (Table 4). When the ${r}_{a}$ assessed for CSE and ALP separately, the most striking finding was the very high unfavourable correlation obtained between DBH21 and MOE for ALP (-0.83 ± 0.18) compared to CSE (-0.44 ± 0.12). Contrarily, ${r}_{a}$ between density and growth-related properties (i.e., between DENS and RWT) were less unfavourable in ALP (-0.54 ± 0.12), compared to CSE (-0.66 ± 0.06) (Table 5).

**Table 4:** Additive genetic correlation estimates among growth and wood properties based on model-A and model-B. (Standard errors of the estimates in the parentheses)

model-A					model-B				
Trait	DBH21	DENS	CANDENS	CELL	Trait	DBH21	DENS	CANDENS	CELL
RWT	0.98 (0.01)	-0.74 (0.03)	-0.38 (0.08)	-0.24 (0.11)	RWT	0.96 (0.02)	-0.62 (0.05)	-0.13 (0.11)	0.18 (0.13)
MOE	-0.66 (0.05)	0.90 (0.02)	0.31 (0.09)	0.60 (0.08)	MOE	-0.52 (0.09)	0.88 (0.03)	0.11 (0.10)	0.60 (0.08)
TRadW	0.54 (0.05)	-0.65 (0.03)	-0.22 (0.08)	-0.23 (0.11)	TRadW	0.51 (0.07)	-0.58 (0.05)	-0.08 (0.09)	-0.19 (0.11)
TWTH	-0.53 (0.06)	0.92 (0.01)	0.17 (0.09)	0.57 (0.10)	TWTH	-0.37 (0.09)	0.90 (0.01)	0.00	0.59 (0.10)
NUMRES	0.43 (0.08)	-0.50 (0.07)	0.49 (0.08)	-0.15 (0.14)	NUMRES	0.40 (0.11)	-0.42 (0.08)	0.70 (0.05)	-0.13 (0.14)
LIG	0.32 (0.11)	-0.62 (0.08)	-0.16 (0.12)	-0.98 (0.03)	LIG	0.23 (0.14)	-0.60 (0.08)	-0.08 (0.13)	-0.98 (0.03)

**Table 5:** Additive genetic correlation estimates among growth and wood properties based on northern (CSE) and southern (ALP) populations. (Standard errors of the estimates in the parentheses)

CSE					ALP				
Trait	DBH21	DENS	CANDENS	CELL	Trait	DBH21	DENS	CANDENS	CELL
RWT	0.96 (0.03)	-0.66 (0.06)	-0.05 (0.20)	-0.13 (0.17)	RWT	0.99 (0.04)	-0.54 (0.12)	-0.06 (0.22)	-0.42 (0.25)
MOE	-0.44 (0.12)	0.91 (0.04)	0.06 (0.06)	0.60 (0.12)	MOE	-0.83 (0.18)	0.77 (0.10)	0.12 (0.24)	0.66 (0.17)
TRadW	0.66 (0.07)	-0.65 (0.05)	-0.10 (0.12)	-0.19 (0.20)	TRadW	0.33 (0.18)	-0.38 (0.13)	0.05 (0.10)	-0.04 (0.12)
TWTH	-0.34 (0.11)	0.91 (0.01)	-0.01 (0.10)	0.45 (0.14)	TWTH	-0.17 (0.21)	0.82 (0.05)	0.13 (0.21)	0.82 (0.21)
NUMRES	0.54 (0.12)	-0.47 (0.10)	0.69 (0.08)	-0.18 (0.20)	NUMRES	0.32 (0.22)	-0.38 (0.16)	0.66 (0.12)	-0.36 (0.27)
LIG	0.17 (0.19)	-0.59 (0.13)	0.04	-0.95 (0.04)	LIG	0.40 (0.28)	-0.67 (0.18)	0.02 (0.12)	-0.99 (0.05)

As expected, Type-B genetic correlations across sites (${r}_{b}$), representing the level of genotype by environment interaction (G × E) was low for wood properties, with ${r}_{b}$ ranging from 0.79 to 0.92 for solidwood and tracheid properties and from 0.91 to 0.99 for resin properties. Furthermore, ${r}_{b}$ across sites were very high for growth properties in this study (${r}_{b}$~ 0.99 for HI, DBH12, and DBH21) based on both models, except for the lower correlation obtained for RWT (${r}_{b}$=0.77 and 0.60) based on model-A and model-B, respectively. However, genetic correlations between sites for chemical properties were much lower, (${r}_{b}$ ~ 0.45 for CELL and HEM, and 0.72 for LIG) (Table S2). Overall, model-B resulted in a slight, but negligible, reduction of ${r}_{b}$ for the studied traits, compared to model-A.

## Discussion

It has been demonstrated how population and family structure can adversely affect accuracies of genomic predictions and response to selections in practical plant breeding schemes [28]. Likewise, incorporation of contemporary groups implemented directly in the pedigree or in the model as a fixed or random effect, was shown to improve model fitting and accuracy of breeding values in Douglas-fir [29]. To our knowledge, this is the first quantitative genetic study to extensively examine the effect of marker-based population structure in quantitative genetic evaluations for unbiased estimation of genetic parameters, utilizing a large data set retrieved from the Swedish Norway spruce breeding program.

In the current study, significant differences in phenotypic and genetic performances of populations were observed, except for chemical properties. Additionally, across-population variation in estimated breeding values (EBVs) of individuals was notably higher than those observed for phenotypes, indicating differences among populations is due to genetic factors. Trees originating from southern latitudes, for instance those from CEU and ALP, were taller and larger than trees originating from northern latitudes, when grown in southern Sweden. Such result is in line with previous reports and the practical breeding guidelines for Norway spruce, which indicates that trees from southern origins outperform local provenances [18, 30]. Previously, it has been suggested that trees transferred to a higher latitude take advantage of longer photoperiods during growing season, and thereby have a higher growth rate than the local trees [31]. Contrarily, trees originating from higher latitudes, such as CSE and RusBal had higher wood density, supporting the unfavourable association between low wood density and fast growth [32]; cheaper stem construction (low wood density) will increase the rate of leaf deployment per total mass increment and therefore growth rate in height and diameter [33]. The among-population genetic differentiation (Q_ST_) for all studied traits was found to be highest for growth traits, especially at later ages (RWT, DBH12, DBH21), and secondly for solid wood properties (DENS, MOE, PILOD, and MFA). This implies a high level of local adaptive potential in traits related to productivity [34]. Nonetheless, Q_ST_ values were much lower for tracheid and resin properties, and mostly negligible for chemical properties. This is in accordance with one recent study that investigated population differentiation and adaptation of Norway spruce for different traits by comparing Q_ST_ and F_ST_ [7]. The authors showed that the macroscopic trait of stem diameter, in contrast to its microscopic components such as tracheid dimensions, is subject to divergent selection that dates back before the Last Glacial Maximum. Similarly, another study reported Q_ST_ values being the highest for DBH in coastal Douglas-fir [29].

The primary goal in every tree improvement program is to maximize genetic gain in economically important traits, which can be estimated as a product of heritability and selection differential [12]. In our study, narrow sense heritability estimates (${h}^{2}$) obtained for growth and wood density were significantly larger based on the model ignoring the effect of population structure (model-A) than those obtained based on the model incorporating population effect (model-B). When partitioning the estimated genetic variance into its components, we have observed that such upward bias in model-A is mostly absorbed by the additive genetic variance. We reason that model-B allows dissection of the total genetic variation into components that reside within and among populations, whereas in model-A the additive genetic variation within and among-populations are confounded. This implies that failing to account for the among-population variation as a separate effect, particularly for traits having high Q_ST_, such as growth-related properties in the current investigation, can result in overestimation of the additive genetic variance. This ultimately leads to the biased prediction of the response to selection if the selection is carried out within populations or even within families. Nevertheless, when the focus is placed on selection of the best model, most of these decisions are made towards enhancement of predictive accuracy, which is a measure of the reliability of EBVs and is used to predict the response to selection [35]. This accuracy can in practice be estimated by means of cross validations [36]. Among others, random cross-validation is a common approach to assess efficiencies or accuracies of the estimated predictions in breeding schemes. Throughout the work we used 5-fold cross-validations to compare the models, measured as the correlation between predictions and observations. Model-B resulted in considerably higher predictive ability and prediction accuracy, particularly for growth and solidwood properties. It is noteworthy to mention that cross-validation results are consistent for both sites, thus supporting the notion that the appropriate modelling of population structure in genetic evaluations has practical implications on the outcome of the selection process, and in turn, breeding program of Norway spruce.

Genetic correlations measure the level of relationship between two traits owing to genetic causes. Negative genetic correlations among desirable traits are often used as evidence for trade-offs and their investigation is important in understanding the evolutionary response of a trait as well as in designing effective breeding programs [37]. One of the major causes of trade-offs is antagonistic pleiotropy, i.e., alleles that give rise to a high value for one trait and a low for the other. Another possible cause of trade-offs, although transient, is gametic phase linkage disequilibrium, which may occur when individuals from two populations with different gene frequencies intermate, as a side effect of recent directional selection or by biased or limited sampling [38]. Nevertheless, genetic correlation is a complex population-specific genetic parameter, subject to be influenced by both allele frequency and environmental changes [37].

In line with previous reports in several coniferous species [39], additive genetic correlation estimates obtained for DENS and MOE with growth traits were negative across the models, indicating breeding for increased volume is achievable at the cost of decreased stem quality. Similarly, additive genetic correlation of lignin with density-related traits was unfavourable across the tested models, suggesting simultaneous improvement of trees for enhanced wood-quality and bioenergy production remains challenging in Norway spruce. Although the pattern of correlations was similar across the models, their magnitude was slightly lower in model-B, suggesting that selections consistently performed within populations may suffer less from the growth-wood quality trade-offs, compared to mass selections conducted without pedigree restrictions. More specifically, additive genetic correlations obtained in model-A are inflated as their variance is confounded with the variation residing among populations, and therefore, disentanglement of these effects in model-B resulted in the reduction of correlation estimates.

Our results similarly demonstrated that the magnitude of correlations can vary among populations. For instance, the unfavourable genetic correlation of growth with wood stiffness obtained for northern population (CSE) was only 50% of the one found for southern population (ALP), while the negative association of growth with wood density was weaker in ALP, compared to CSE.

Results of type-B genetic correlations revealed no evidence of genotype by environment interaction (G × E) for growth and wood properties underlying this study. However, type-B correlations were moderate to low for chemical properties, an indication of low stability in the performances of genotypes across the two sites for these traits. In general, there are various spectroscopic techniques, such as near-infrared spectroscopy (NIR), that can be successfully applied for rapid assessment of “hard-to-measure” chemical composition of wood [40]. Although such techniques are highly advantageous, one of their drawbacks is that the predicted relationship between wavelengths and the property of interest are based only on a subset of samples, derived from wet chemistry analyses [41] like the technique used in the current study. Correspondingly, the poor performance of chemical properties, in terms of non-significant differences observed across populations, very low Q_ST_ estimates, and high levels of G × E obtained in our study could be due to some extra noise affected prediction of the chemical properties. Future investigations should examine other spectroscopical methods along with different prediction and calibration models to determine whether the non-significant differences observed among populations for chemical properties has a biological or technical underlying factor.

## Conclusions

In the current study, we examined the impact of marker-based population structure on genetic parameter estimates, utilizing a large data set retrieved from the breeding program of Norway spruce in Sweden, through two alternative models. Our findings show that there is a substantial genetic variation among populations, in terms of growth and wood properties. The model which accounts for population structure as a separate effect (model-B) results in substantial reduction of additive genetic variance, and subsequently, reduction of narrow-sense heritability estimates. However, prediction accuracies obtained based on this model were considerably higher than the alternative model (model-A). This was particularly significant for growth and solid-wood properties, which showed to have the highest population genetic differentiation (Q_ST_) among the studied traits. Although the adverse genetic correlation between growth and wood properties remains as a constraint, the magnitude of correlations was slightly lower in model-B. This suggests that selections consistently performed within populations may suffer less from the growth-wood quality trade-offs, compared to mass selections conducted without restrictions in terms of population-pedigree. Along with the lower accuracies obtained based on model-A, we may conclude that results of models neglecting effect of population structure are inaccurate and biased as the variation residing among populations is confounded with the additive genetic variance. This might have practical implications for future Norway spruce breeding program and potentially for other species when the breeding materials have heterogenous background.

## Materials and methods

### Experimental materials

This study utilized data from two large open-pollinated progeny trials of Norway spruce: S21F9021146 (Höreda) (57.61°N 15.04°E) and S21F9021147 (Erikstorp) (55.90°N 13.93°E), located in southern Sweden. Both trials were established in the spring of 1990 by the Forestry Research Institute of Sweden (Skogforsk) and are a part of the breeding program of Norway spruce in southern Sweden. The genetic material in each trial originates from open-pollinated seeds collected from standing plus-trees (trees with outstanding phenotype) within 112 stands. Each experiment has a randomized incomplete block with single-tree plot design, divided into 20 and 23 blocks, comprised of 1,373 and 1,375 half-sib families, in S21F9021146 and S21F9021147, respectively. More detailed information about trial characteristics is found in [26].

### Phenotype measurements

Eighteen traits related to growth, wood quality (solid and tracheid properties), chemical composition and resin canal properties of wood were assessed in this study. Tree diameter at breast height (1.3 m above ground) was measured at ages 12 and 21 years (DBH12 and DBH21 [mm], respectively) and tree height was measured at age seven (HI7 [cm]). Trees were also assessed for pilodyn penetration at age 22 years (PILOD [mm]), which offers an indirect measure of wood density [42]. A complete list of the measured traits, their abbreviations, and number of individuals and families representing them, is shown in Table 6.

**Table 6:** Summary of traits measured for this study

Measured Traits	Traits category	Abbreviations(unit)	No. Families	No. Trees
Diameter_age 12	Growth	DBH12 (mm)	1370	9201
Diameter_age 21	Growth	DBH21 (mm)	1370	7784
Height_age 7	Growth	HI7 (cm)	1370	9338
Annual Ring width	Growth	RWT (mm)	524	5661
Pilodyn penetration	Solidwood	PILOD (mm)	524	5617
Density	Solidwood	DENS (kg/m3)	524	5617
Modulus of elasticity	Solidwood	MOE (Gpa)	524	5609
Microfibril angle	Solidwood	MFA (degree)	524	5617
Tracheid radial width	Tracheid	TRadW (μm)	524	5617
Tracheid tangential width	Tracheid	TTangW(μm)	524	5617
Tracheid wall thickness	Tracheid	TWTH (μm)	524	5617
Tracheid coarseness	Tracheid	TC (µg/m)	524	5617
Number of Resin canals	Resin	NUMRES	524	4873
Average area of resin canals	Resin	AVCAREA (μm^2^)	524	4873
Density of Resin canals	Resin	CANDENS (canal/cm^2^)	524	4873
Cellulose	Chemical	CELL (%)	524	3822
Hemicellulose	Chemical	HEM (%)	524	3818
Lignin	Chemical	LIG (%)	524	3822

### SilviScan measurements

In 2010 and 2011, two 12-mm bark-to-pith increment cores were collected for analyses of radial variations of different traits at breast height from 5,666 trees, aged 20–21 years, representing 524 half-sib families. The cores were drilled from the northern side of the stems, in parallel and close to each other to allow joint evaluations of property data origination from the two cores. The same core was used for radial analyses of growth, solid, tracheid and chemical properties, all performed at Innventia, now RISE (Stockholm, Sweden).

High-resolution data were acquired with a SilviScan instrument [43] on pith-to-bark radial variations on traits important for solidwood properties (wood density (DENS [kg/m^3^]), modulus of elasticity (MOE [GPa]), microfibril angle (MFA [degree])); tracheid properties (radial tracheid width (TRadW [μm]), tangential tracheid width (TTangW [μm]), tracheid wall thickness (TWTH [µm]), tracheid coarseness (TC [µg/m])); and ring width (RWT [mm]). The three ring segments, earlywood (EW), transition wood (TW) and latewood (LW), were identified from the wood density variation within each ring [44].

Because area-weighted values (AWV) more accurately represent the average properties of the wood the AWV for solid and tracheid properties was calculated and used in this study [45].

### Chemical wood properties

Models for estimation of concentrations of wood chemical components (lignin (LIG), cellulose (CELL), and hemicelluloses (HEM) [%]) were developed from wood sampled from trees of similar origin. A set of 40 annual rings selected with the aim of covering as much chemical variability in the wood as possible in the chemical concentrations was identified and cut out from the discs with one longitudinal x radial surface of same orientation as the sides of the SilviScan sample strips. These sides, facing the side of the tracheid, were scanned with a hyperspectral imaging near infrared camera (NIR, 960 – 2500 nm), providing spectra with 6 nm spectral resolution, and mean spectra for each sample (ring) were calculated. The samples were then analysed with chemical reference methods at MoRe Research (Örnsköldsvik, Sweden). The carbohydrates and lignin content for all samples were analysed by the SCAN-CM 71:09 and Tappi T222 methods [46], respectively. With the SCAN-CM 71:09 method, the different sugar monomers were obtained and from the monomer content, the percentage of cellulose and hemicellulose were quantified, following the formula developed for softwoods [47]. The chemical ring mean data were associated with corresponding NIR spectra and partial least squares multivariate models for estimation of the chemical concentrations were created and validated with the data. The SilviScan samples were then polished on the corresponding sides and scanned with the same NIR-camera at 30 µm radial resolution, and the variation in chemical composition was predicted for all samples. The chemical composition at each point in the sample was spatially matched to the physical characteristics as determined by SilviScan, using an algorithm designed for the purpose, which allowed calculating ring-mean averages of the chemical composition. The coefficient of determination (R^2^) and root mean square error of cross-validation (RMSEcv) for each model are given in Table S3.

### Resin wood properties

Identification of the resin properties of wood was done using a learning-based model approach. Commonly, about 60 individual microscopy images are needed to cover a single SilviScan sample. A random sample of 104 high-resolution images obtained from different SilviScan analysis, were used to annotate, train, and test a neural network model to count the total number of resin canals (NUMRES) in each single microscopy image and the area of every resin canal, using the STARDIST method [48]. To validate the performance of the model, it was deployed to predict the number of resin canals in 1634 individual microscopy images, corresponding to 25 SilviScan samples that were not used for training. The actual number of resin canals in these 25 samples was also manually counted and compared with the model’s predictions, giving a coefficient of determination (R^2^) of 0.8905. The model was then applied to the samples in this study. Subsequently, resin canal density (CANDENS [canal/cm^2^]) of each sample was determined as NUMRES per unit area, while the average crosscut area of the resin canals (AVCAREA [μm^2^]) was determined by dividing the total area of the resin canals by NUMRES.

### Population structure based on DNA markers

Population structure of the individuals derived from SNP genotyping of the 518 mother trees. The analysis of population structure was applied on 399,801 noncoding SNPs with significantly linked sites removed (pairwise LD ≥ 0.2 and FDR value ≤ 0.05). EIGENSOFT v6.1.4 was used to perform principal component analysis (PCA) on the reduced set of independent SNPs. Individuals of known origin were first grouped into seven major clusters. These individuals were then used as a training set in a ‘Random Forest’ regression model. The first five components of the PCA analysis were used for model fitting to classify the unknown individuals into each of the seven clusters. Fivefold cross‐validation was performed for error estimation [6, 22]. The half-sib families are categorized into seven genetic clusters as follows: Central and South Sweden (CSE), Russia-Baltics (RusBal), Northern Poland (NPL), Central Europe (CEU), Alpine (ALP), Carpathians (ROM), and hybrids between CSE and ALP (CSE-ALP). The individuals belonging to the ROM cluster were excluded from the analysis, because this cluster was represented only by three half-sib families, whereas all other clusters comprised at least 10 families (Table S4).

### Quantitative genetic parameters

Population structure can be fitted as either fixed or random effect in genetic evaluations using mixed-linear models [49]. In the current study, population structure was considered as a fixed term, although alternative attempts to model it as random term or as an intrinsic part of the pedigree yielded similar results (data not presented). The effect of population structure on estimated genetic parameters as well as estimated breeding values (EBVs) was investigated through two alternative models 1) population structure excluded from the model entirely (model-A) 2) all populations included in the model (model-B). Later, the accuracy and predictive performance of these two models were compared using a k-fold cross-validation based on all studied traits.

Variance and covariance components for the studied traits were estimated using univariate and bivariate mixed linear models implemented in the ASReml-R statistical package [50]. The fit of different models was evaluated using the Akaike Information Criteria (AIC) and loglikelihood estimates and the optimal model was selected based on a compromise of model fit and complexity. The following pedigree-based linear mixed (animal) model for joint-site analysis of model-A and model-B was fitted, with the only difference that in model-A the effect of population structure was dropped from the model.

1 $$Y_{jklm}=\mu+P_l{+S}_j{+B}_{k(j)}+G_{m(l)}+SG_{jm(l)}+e_{jklm}$$

where $$Y$$ is the vector of observations on tree $$m$$ from genetic cluster (population) $$l$$ in block $$k$$ at the site $$j$$, µ is overall mean,$${P}_{l}$$,$${S}_{j}$$, and $${B}_{k(j)}$$ are the fixed effects of population$$l$$, site $$j$$, and block $$k$$ within the site$$j$$, respectively. The variables $${G}_{m(l)}$$ and $$S{G}_{jm(l)}$$ are the random additive genetic effects of individual $$m$$ within population$$l$$, and the random interactive effect of the site $$j$$ and the individual $$m$$ within the population$$l$$, respectively, and $${e}_{jklm}$$ is the random residual effect. Preliminary analyses indicated there was no significant population-by-site ($$S{P}_{jl})$$ effect for all traits, except for DBH12 and PILOD. Therefore, this effect was omitted from the model.

For the analysis and estimation of type-B genetic correlation between sites, heterogeneous additive genetic variance $\sim MVN(0,G\otimes I)$ and heterogenous error variance$\sim MVN(0,R\otimes I)$ were included in the model.

$$G=\begin{bmatrix}\sigma_{a1}^2&\sigma_{a1a2}\\\sigma_{a2a1}&\sigma_{a2}^2\end{bmatrix}\;R=\begin{bmatrix}\sigma_{e1}^2&0\\0&\sigma_{e2}^2\end{bmatrix}$$ where $\sigma_{ai}^2$ and $\sigma_{ai}$ are the additive genetic variances and covariances, respectively; $\sigma_{ei}^2$ is the error variance for each site; $I$ is the identity matrix equal to the number of observations at each site and 0 indicates no site–site error covariance.

Breeding values of individuals for model-B were calculated using genetic effect estimates obtained from equation one, as follows:

2 $${EBV}_{(lk)}=\widehat{\mu }+{\widehat{G}}_{m(l)}+{\widehat{P}}_{l}$$

where $\widehat{\mu },\widehat{G},\text{ and} \widehat{P}$ are predicted mean, solutions of individual tree and population effects, respectively. However, for model-A, the effect of population was not included.

For estimating type-A genetic correlation between different traits, bivariate analysis was conducted featuring a similar model setup as in Eq. 1, except that the genetic variance was considered as homogenous.

The narrow-sense heritability (${h}_{i}^{2}$) for each trait, using variance components obtained from Eq. 1, was calculated as:

3 $${h}_{i}^{2}=\frac{{\widehat{\sigma }}_{A}^{2}}{{\widehat{\sigma }}_{P}^{2}}$$

where ${\widehat{\sigma }}_{A}^{2}$, ${\widehat{\sigma }}_{p}^{2}$ are additive genetic and phenotypic variance components, respectively.

The additive genetic correlations between traits ${a}_{1}$ and ${a}_{2}$ (${r}_{(a)}$), using variance and covariance components from the bivariate analysis, were calculated as:

4 $${r}_{(a)}=\frac{{\widehat{\sigma }}_{a\text{1,2}}}{\sqrt{ {\widehat{\sigma }}_{a1}^{2} \times {\widehat{\sigma }}_{a2}^{2}}}$$

Type-B genetic correlations [51] of additive effects across sites, were calculated as follows:

5 $${r}_{(B)}=\frac{{\widehat{\sigma }}_{a\text{1,2}}}{\sqrt{ {\widehat{\sigma }}_{a1}^{2} \times {\widehat{\sigma }}_{a2}^{2}}}$$

where ${\sigma }_{a\text{1,2}}$ is the covariance between the additive effects of the same trait at different sites; ${\sigma }_{a1}^{2}$ and ${\sigma }_{a2}^{2}$ are estimated additive variances for the same traits at different sites. Standard errors for variance components and genetic parameters were estimated using the Taylor series expansion method.

Population differentiation of quantitative traits, represented by ${Q}_{st}$ index [52], was estimated following the mixed model (Eq. 1), except that effect of genetic cluster was considered as random and ${Q}_{st}$ was calculated as:

6 $${Q}_{ST}=\frac{{\widehat{\sigma }}_{G}^{2}}{{\widehat{\sigma }}_{G}^{2}+2{\widehat{\sigma }}_{A}^{2}}$$

where ${\sigma }_{G}^{2}$ is the variance of genetic cluster and other terms were defined above in Eq. 3.

### Cross-validation for comparison of model performance

In this work we used a five-fold cross validation for all traits to compare predictive performance of the two model alternatives (model-A and model-B). The procedure consisted of dividing the dataset into five random folds of approximately equal size. Data in four folds were used for training the model (model development) and prediction of phenotypes in the one remaining fold (the testing fold) that has the phenotypes set to missing. The prediction process was repeated five times until each fold had been used for once as test set. The performance of the models was assessed by predictive ability and prediction accuracy. Predictive ability (PA) was evaluated as the mean Pearson correlation of estimated breeding values (EBVs) of the individuals from the five replications with their observed phenotype ($y$). i.e., $PA=cor\left(EBVs, y\right)$. Standard error of the correlations was computed using the following equation:

7 $$SE=\frac{\sigma }{\sqrt{n}}$$

where $\sigma$ is the standard deviation of the Pearson correlations and n is the number of replicates. Additionally, ACC was estimated as the PA scaled by average square root of heritabilities obtained from each replication. i.e., $\text{ACC}=\frac{\text{PA}}{\sqrt{{\text{h}}_{\text{i}}^{2}}}$.

### Supplementary Information

Supplementary Material 1.

Supplementary Material 2.

## Data Availability

The phenotypic datasets used and/or analysed during the current study are available from the corresponding author on reasonable request. The exome capture raw reads and the RNA-seq data have been deposited in NCBI's sequence read archive (SRA) under accession number PRJNA511374 (Chen et al., 2019) and PRJNA731384 (Chen et al., 2021).

## Abbreviations

PA: Predictive ability
ACC: Prediction accuracy
EBVs: Estimated breeding values
G × E: Genotype by environment interaction
AWV: Area-weighted values
PCA: Principal component analysis
NFE: Northern Fennoscandia
CSE: Central and South Sweden
RusBal: Russia-Baltics
NPL: Northern Poland
CEU: Central Europe
ALP: Alpine
ROM: Carpathian
RWT: Annual ring width
HI7: Height measured at age 7
DBH12: Diameter at breast height age 12
DBH21: Diameter at breast height age 21
PILOD: Pilodyn penetration
TWTH: Tracheid wall thickness
TC: Tracheid coarseness
TRadW: Radial tracheid radial width
TTangW: Tracheid tangential width
MOE: Modulus of elasticity
DENS: Wood density
MFA: Microfibril angle
NUMRES: Number of resin canals
AVCAREA: Average area of resin canals
CANDENS: Density of resin canals
CELL: Cellulose content
HEM: Hemicellulose content
LIG: Lignin content

## References

[1] Bennett K (1997). Evolution and Ecology: the Pace of Life.

[2] Petit RJ, Aguinagalde I, de Beaulieu J-L, Bittkau C, Brewer S, Cheddadi R (2003). Glacial refugia: hotspots but not melting pots of genetic diversity. Science.

[3] García-Gil MR, Mikkonen M, Savolainen O (2003). Nucleotide diversity at two phytochrome loci along a latitudinal cline in Pinus sylvestris. Mol Ecol.

[4] Mølmann JA, Junttila O, Johnsen Ø, Olsen JE (2006). Effects of red, far-red and blue light in maintaining growth in latitudinal populations of Norway spruce (Picea abies). Plant, Cell Environ.

[5] Saccheri I, Hanski I (2006). Natural selection and population dynamics. Trends Ecol Evol.

[6] Milesi P, Berlin M, Chen J, Orsucci M, Li L, Jansson G (2019). Assessing the potential for assisted gene flow using past introduction of Norway spruce in southern Sweden: Local adaptation and genetic basis of quantitative traits in trees. Evol Appl.

[7] Tiret M, Olsson L, Grahn T, Karlsson B, Milesi P, Lascoux M (2023). Divergent selection predating the Last Glacial Maximum mainly acted on macro-phenotypes in Norway spruce. Evol Appl.

[8] Gienapp P, Teplitsky C, Alho J, Mills J, Merilä J (2008). Climate change and evolution: disentangling environmental and genetic responses. Mol Ecol.

[9] Root TL, Price JT, Hall KR, Schneider SH, Rosenzweig C, Pounds JA (2003). Fingerprints of global warming on wild animals and plants. Nature.

[10] Stojanova B, Koláříková V, Šurinová M, Klápště J, Hadincová V, Münzbergová Z (2019). Evolutionary potential of a widespread clonal grass under changing climate. J Evol Biol.

[11] Falconer DS, Mackay TF (1983). Quantitative genetics.

[12] White TL, Adams WT, Neale DB (2007). Forest genetics.

[13] O’Reilly-Wapstra JM, Miller AM, Hamilton MG, Williams D, Glancy-Dean N, Potts BM (2013). Chemical variation in a dominant tree species: population divergence, selection and genetic stability across environments. PLoS ONE.

[14] Matyas C (1996). Climatic adaptation of trees: rediscovering provenance tests. Euphytica.

[15] George JP, Theroux‐Rancourt G, Rungwattana K, Scheffknecht S, Momirovic N, Neuhauser L (2020). Assessing adaptive and plastic responses in growth and functional traits in a 10‐year‐old common garden experiment with pedunculate oak (Quercus robur L.) suggests that directional selection can drive climatic adaptation. Evolutionary Applications.

[16] Hannrup B, Cahalan C, Chantre G, Grabner M, Karlsson B, Bayon IL (2004). Genetic parameters of growth and wood quality traits in Picea abies. Scand J For Res.

[17] Karlsson B, Rosvall O, editors. Breeding programmes in Sweden: Norway spruce. Proceedings of progeny testing and breeding strategies Meeting of the Nordic Group for Tree Breeding, Edinburgh; 1993.

[18] Persson A, Persson B. Survival, growth and quality of Norway spruce (Picea abies (L.) Karst.) provenances at the three Swedish sites of the IUFRO 1964/68 provenance experiment. Rapport-Sveriges Lantbruksuniversitet. 1992.

[19] Myking T, Rusanen M, Steffenrem A, Kjær ED, Jansson G (2016). Historic transfer of forest reproductive material in the Nordic region: drivers, scale and implications. Forestry: An International Journal of Forest Research.

[20] Källman T (2009). Adaptive evolution and demographic history of Norway spruce (Picea abies).

[21] Vendramin G, Anzidei M, Madaghiele A, Sperisen C, Bucci G (2000). Chloroplast microsatellite analysis reveals the presence of population subdivision in Norway spruce (Picea abies K.). Genome.

[22] Chen J, Li L, Milesi P, Jansson G, Berlin M, Karlsson B (2019). Genomic data provide new insights on the demographic history and the extent of recent material transfers in Norway spruce. Evol Appl.

[23] Chen Z-Q,  Zan Y, Milesi P, Zhou L, Chen J, Li L (2021). Leveraging breeding programs and genomic data in Norway spruce (Picea abies L. Karst) for GWAS analysis. Genome biology.

[24] Li L, Milesi P, Tiret M, Chen J, Sendrowski J, Baison J (2022). Teasing apart the joint effect of demography and natural selection in the birth of a contact zone. New Phytol.

[25] Steffenrem A, Solheim H, Skrøppa T (2016). Genetic parameters for wood quality traits and resistance to the pathogens Heterobasidion parviporum and Endoconidiophora polonica in a Norway spruce breeding population. Eur J Forest Res.

[26] Chen Z-Q, Gil MRG, Karlsson B, Lundqvist S-O, Olsson L, Wu HX (2014). Inheritance of growth and solid wood quality traits in a large Norway spruce population tested at two locations in southern Sweden. Tree Genet Genomes.

[27] Chen Z-q (2016). Quantitative genetics of Norway spruce in Sweden.

[28] Werner CR, Gaynor RC, Gorjanc G, Hickey JM, Kox T, Abbadi A (2020). How population structure impacts genomic selection accuracy in cross-validation: implications for practical breeding. Front Plant Sci.

[29] Klápště J, Suontama M, Dungey HS, Telfer EJ, Stovold GT (2019). Modelling of population structure through contemporary groups in genetic evaluation. BMC Genet.

[30] Liziniewicz M, Berlin M, Solvin T, Hallingbäck HR, Haapanen M, Ruotsalainen S (2023). Development of a universal height response model for transfer of Norway spruce (Picea abies L. Karst) in Fennoscandia. Forest Ecology and Management.

[31] Dormling I, editor Influence of light intensity and temperature on photoperiodic response of Norway spruce provenances. Proc IUFRO Norway spruce Meeting, Bucharest; 1979.

[32] Poorter L, McDonald I, Alarcón A, Fichtler E, Licona JC, Peña-Claros M (2010). The importance of wood traits and hydraulic conductance for the performance and life history strategies of 42 rainforest tree species. New Phytol.

[33] Gibert A, Gray EF, Westoby M, Wright IJ, Falster DS (2016). On the link between functional traits and growth rate: meta-analysis shows effects change with plant size, as predicted. J Ecol.

[34] Savolainen O, Pyhäjärvi T, Knürr T (2007). Gene flow and local adaptation in trees. Annu Rev Ecol Evol Syst.

[35] Bijma P (2012). Accuracies of estimated breeding values from ordinary genetic evaluations do not reflect the correlation between true and estimated breeding values in selected populations. J Anim Breed Genet.

[36] Schrauf MF, de Los CG, Munilla S (2021). Comparing genomic prediction models by means of cross validation. Front Plant Sci.

[37] Wu HX, Sanchez L (2011). Effect of selection method on genetic correlation and gain in a two-trait selection scheme. Aust For.

[38] Sánchez L, Yanchuk AA, King JN (2008). Gametic models for multitrait selection schemes to study variance of response and drift under adverse genetic correlations. Tree Genet Genomes.

[39] Wu H, Ivkovic M, Gapare W, Matheson A, Baltunis B, Powell M (2008). Breeding for wood quality and profit in Pinus radiata: a review of genetic parameter estimates and implications for breeding and deployment. NZ J Forest Sci.

[40] Cozzolino D (2014). Use of infrared spectroscopy for in-field measurement and phenotyping of plant properties: instrumentation, data analysis, and examples. Appl Spectrosc Rev.

[41] Yun Y-H, Li H-D, Deng B-C, Cao D-S (2019). An overview of variable selection methods in multivariate analysis of near-infrared spectra. TrAC, Trends Anal Chem.

[42] Chen Z-Q, Karlsson B, Lundqvist S-O, García Gil MR, Olsson L, Wu HX (2015). Estimating solid wood properties using Pilodyn and acoustic velocity on standing trees of Norway spruce. Ann For Sci.

[43] Evans R (1994). Rapid measurement of the transverse dimensions of tracheids in radial wood sections from Pinus radiata. Holzforschung.

[44] Lundqvist S-O, Seifert S, Grahn T, Olsson L, García-Gil MR, Karlsson B (2018). Age and weather effects on between and within ring variations of number, width and coarseness of tracheids and radial growth of young Norway spruce. Eur J Forest Res.

[45] Gräns D, Hannrup B, Isik F, Lundqvist S-O, McKeand S (2009). Genetic variation and relationships to growth traits for microfibril angle, wood density and modulus of elasticity in a Picea abies clonal trial in southern Sweden. Scand J For Res.

[46] Standard T. Acid-insoluble lignin in wood and pulp. T222 om-02. 2002.

[47] Sjöström E. Wood chemistry: fundamentals and applications. San Diego, CA: Academic Press.: Elsevier; 1993.

[48] Schmidt U, Weigert M, Broaddus C, Myers G, editors. Cell detection with star-convex polygons. Medical Image Computing and Computer Assisted Intervention–MICCAI 2018: 21st International Conference, Granada, Spain, September 16–20, 2018, Proceedings, Part II 11; 2018: Springer.

[49] Ugarte E, Alenda R, Carabano M (1992). Fixed or random contemporary groups in genetic evaluations. J Dairy Sci.

[50] Butler D, Cullis BR, Gilmour A, Gogel B. ASReml-R reference manual. The State of Queensland, Department of Primary Industries and Fisheries, Brisbane. 2009.

[51] Burdon RD (1977). Genetic correlation as a concept for studying genotype-environment interaction in forest tree breeding.

[52] Spitze K (1993). Population structure in Daphnia obtusa: quantitative genetic and allozymic variation. Genetics.

