# Correction: Implications of accounting for marker-based population structure in the quantitative genetic evaluation of genetic parameters related to growth and wood properties in Norway spruce

**DOI:** 10.1186/s12863-024-01250-w

**Published:** 2024-07-03

**Authors:** Haleh Hayatgheibi, Henrik R. Hallingbäck, Sven-Olof Lundqvist, Thomas Grahn, Gerhard Scheepers, Peter Nordström, Zhi-Qiang Chen, Katri Kärkkäinen, Harry X. Wu, M. Rosario García-Gil

**Affiliations:** 1 Department of Forest Genetics and Plant Physiology, Swedish University of Agricultural Sciences (SLU), Umeå, Sweden; 2 Forestry Research Institute Sweden (Skogforsk), Uppsala, 75183 Sweden; 3 IIC, Stockholm, Sweden; 4 RISE AB, Stockholm, Sweden; 5 RISE AB, Växjö, Sweden; 6 RISE AB, Umeå, Sweden; 7 Natural Resources Institute Finland (LUKE), OULU, Finland; 8 Beijing Advanced Innovation Centre for Tree Breeding By Molecular Design, Beijing Forestry University, Beijing, China; 9 Black Mountain Laboratory, CSIRO National Collection Research Australia, Canberra, Australia

**Correction: BMC Genomic Data (2024) 25:60**

10.1186/s12863-024-01241-x

Following publication of the article, the authors flagged that there was an error in the Background section of the Abstract. Namely, at the end of this section, the following had been written in error: ‘by either incorporating it as a fixed effect (model-A) or excluding it entirely from the analysis (model-B).’ Instead, this should say be as follows: “by either incorporating it as a fixed effect (model-B) or excluding it entirely from the analysis (model-A).”

The published article has since been updated to correct this error. The authors thank you for reading this erratum and apologize for any inconvenience caused.

