# Supplementary material for: Implications of accounting for marker-based population structure in the quantitative genetic evaluation of genetic parameters related to growth and wood properties in Norway spruce
Source: BMC Genom Data. 2024 Jun 14;25:60. doi: 10.1186/s12863-024-01241-x (PMC11177499; doi:10.1186/s12863-024-01241-x)
Supplement: Supplementary file 1 — Supplementary Material 1. [file 12863_2024_1241_MOESM1_ESM.docx]

Supplementary materials

Test statistics results of population structure for different properties

| Table S1. |  |  |
| --- | --- | --- |
| Trait | F-Value | P-Value |
| DBH12 | 40 | < 0.001 |
| DBH21 | 65 | < 0.001 |
| HI7 | 20 | < 0.001 |
| RWT | 58 | < 0.001 |
| PILOD | 19 | < 0.001 |
| DENS | 28 | < 0.001 |
| MOE | 23 | < 0.001 |
| MFA | 9 | < 0.001 |
| TRadW | 11 | < 0.001 |
| Ttang | 7 | < 0.001 |
| TWTH | 20 | < 0.001 |
| TC | 5 | < 0.001 |
| NUMRES | 4.5 | < 0.001 |
| AVCAREA | 3 | 0.025 |
| CANDENS | 8.5 | < 0.001 |
| LIG | 4 | 0.003 |
| CELL | 2 | 0.143 |
| HEM | 1 | 0.532 |

annual ring-width (RWT), diameter measured at ages 21 and 12 (DBH21 and DBH12, respectively), height measured at age 7 (HI7), modulus of elasticity (MOE), density (DENS), pilodyn (PILOD), microfibril angle (MFA), tracheid radial width (TRadW), tangential tracheid width (TTangW), tracheid wall thickness (TWTH), tracheid coarseness (TC), total number of resin canals (NUMRES), average area of resin canals (AVCAREA), resin Canal density (CANDENS), lignin (LIG), Cellulose (CELL) and hemicellulose (HEM) content .

Table S3: Performance metrics of the chemistry models employed in the study.

| Model | R^2^ | RMSEcv |
| --- | --- | --- |
| Cellulose | 0.76 | 1.92 |
| Hemicellulose | 0.79 | 1.40 |
| Lignin | 0.70 | 1.39 |

RMSEcv: root mean square error of cross-validation

 Table S4. Number of half-sib families per genetic cluster.

| Genetic cluster | Number of families |
| --- | --- |
| CSE | 289 |
| RusBal | 28 |
| NPL | 35 |
| CEU | 26 |
| CSE-ALP | 23 |
| ALP | 118 |

Genetic clusters: Central and South Sweden (CSE), Russia-Baltics (RusBal), Northern Poland (NPL), Central Europe (CEU), and hybrids between CSE and ALP (CSE-ALP), Alpines (ALP).

**B)**

**A)**

Fig. S1. Boxplots of phenotypic (A) and genetic (B) (EBVs of individuals obtained based on model-b) performances of Norway spruce populations measured for growth and solidwood properties. Populations ordered by decreasing latitude from left to right on the x-axis. Central and South Sweden (CSE, red), Russia-Baltic (RusBal, olivegreen), Northern Poland (NPL, green), Central Europe CEU, light blue), hybrids between CSE and ALP (CSE-ALP, blue), and Alps (ALP, pink). Trait abbreviations are explained in Table 5 of main text.
